# Transvaginal natural orifice endoscopic surgery for ovarian cystectomy: a more suitable surgical approach for the day-care procedure

**DOI:** 10.3389/fmed.2023.1164970

**Published:** 2023-05-18

**Authors:** Aijie Xie, Xin Li, Juan Huang, Hui Wang, Ying Liu, Lulu Wang, Jianmei Liao, Jie Yu, Ziru Yan, Jiajia Zhang, Liqiong Huang, Tianjiao Liu, Yalan Li, Yonghong Lin, Yujian Jia, Xiaoqin Gan

**Affiliations:** ^1^Chengdu Women's and Children's Central Hospital, School of Medicine, University of Electronic Science and Technology of China, Chengdu, China; ^2^School of Medicine, University of Electronic Science and Technology of China, Chengdu, China; ^3^Department of Gynaecology and Obstetrics, Shanghai Tongren Hospital, Shanghai Jiaotong University School of Medicine, Shanghai, China; ^4^Psychosomatic Medical Center, The Fourth People's Hospital of Chengdu, Chengdu, China

**Keywords:** transvaginal natural orifice endoscopic surgery, ovarian cystectomy, ovarian cyst, transumbilical laparoendoscopic single-site surgery, day-care procedure

## Abstract

**Introduction:**

Although previous studies have shown that vaginal natural orifice transluminal endoscopic surgery (vNOTES) has the advantages of causing less pain, faster recovery, and better concealment of surgical incisions, which aligns with the concept of the day-care procedure, this approach poses a greater risk of damaging adjacent organs (i. e., rectum and bladder) due to its anatomical specificity. Moreover, the day-care procedure may lead to relatively less preoperative evaluation and postoperative care. Hence, it is necessary to explore the safety and effectiveness of vNOTES for ovarian cystectomy in the day-care procedure, to provide a theoretical basis for the wider development of vNOTES surgery.

**Materials and methods:**

This retrospective study included 131 patients at our hospital who underwent ovarian cystectomy from September 2021 to October 2022. Based on the surgical approach, patients were classified into transumbilical laparoendoscopic single-site surgery (LESS) and vNOTES groups. The patients' demographic characteristics and follow-up data were collected during the perioperative period and 1-month postoperatively.

**Results:**

Vaginal natural orifice transluminal endoscopic surgery has less postoperative exhaust time, a lower postoperative 6-hour pain score, and a lower incidence of analgesic drug use, with higher surgical conversion incidence. Multiple linear regression analysis showed that the surgical conversion, chocolate cyst, bilateral cyst, and pelvic adhesion increased the operation duration by ~43 (95% CI: 10.309, 68.152, *p* < 0.001), 15 (95% CI: 6.342, 45.961, *p* = 0.036), 10 (95% CI: 3.07, 40.166, *p* = 0.019), and 8 (95% CI: 4.555, 26.779, *p* = 0.035) min, respectively. Interestingly, vNOTES decreased the operation duration by ~8.5 min (95% CI: −18.313, −2.699, *p* = 0.033).

**Conclusion:**

Vaginal natural orifice transluminal endoscopic surgery was equally safe and effective for ovarian cystectomy compared to LESS. vNOTES aligned with the concept of the day-care procedure due to its reduced postoperative pain, shorter exhaust time, and absence of scarring. However, surgeons should conduct a comprehensive preoperative evaluation and exclude patients suspected to have severe pelvic adhesions.

## Introduction

An ovarian cyst, a common tumor of the female reproductive system, is caused by an abnormal endocrine system or genetic factors (1, 2). It has an incidence rate of 1.3–24.0%, with more than 90% being benign tumors (3, 4). Physiological ovarian cysts generally have no specific symptoms, except for complications, such as torsion and rupture, and do not require any special treatment (5–7). Ovarian cystectomy is often the recommended treatment for symptomatic physiological or pathological ovarian cysts.

With the development of medical devices and the popularization of minimally invasive concepts, patients prefer surgeries that offer better esthetic outcomes, including those that inflict no or smaller scar (8, 9), such as needleoscopic and percutaneous-assisted surgery (10, 11). Currently, the minimally invasive approaches for ovarian cystectomy are vaginal natural orifice transluminal endoscopic surgery (vNOTES) and transumbilical laparoendoscopic single-site surgery (TU-LESS) (12, 13). Meanwhile, enhanced recovery after surgery (ERAS) has developed rapidly, which optimizes the clinical path of perioperative treatment, reduces the stress response of surgical trauma, shortens hospital stay, and promotes rapid recovery of patients (14–16). Based on the ERAS concept, the day-care procedure further optimizes the disease diagnosis and treatment process, which allows patients to be admitted, operated, and discharged within 24 h (17–19). In the two approaches mentioned above, the incisions are hidden in the belly button with no postoperative scar in the vaginal area; thus, they are suitable for the day-care surgery because of their small trauma and quick recovery.

However, although previous studies have shown that vNOTES has the advantages of causing less pain, faster recovery, and better concealment of surgical incisions, this approach poses a greater risk of damaging adjacent organs (i.e., rectum and bladder) due to its anatomical specificity (20–22). Moreover, the day-care procedure may lead to relatively less preoperative evaluation and postoperative care (17, 19). Hence, it is necessary to explore the safety and effectiveness of vNOTES for ovarian cystectomy in the day-care procedure.

Therefore, we investigate the perioperative data of vNOTES for ovarian cystectomy in the day-care procedure and compare them with single-port laparoscopic surgery. The purpose of this study is to explore the safety and effectiveness of vNOTES for ovarian cystectomy in the day-care surgery. In addition, we provide the complete process of the day-care procedure and key points of vNOTES for ovarian cystectomy.

## Materials and methods

### Study design and participants

This study is part of a Longitudinal Vaginal Natural Orifice Transluminal Endoscopic Surgery Study (LovNOTESS) conducted in Chengdu (China Clinical Trials Registry ChiCTR2100053483) and approved by the Ethics Committee of Chengdu Women and Children's Central Hospital (No. 202130). This subgroup study only included the retrospective clinical data of patients with ovarian cysts (i.e., pathological or symptomatic physiological ovarian cysts, with a max diameter of the cyst being >5 cm) who sought surgical treatment in our hospital between September 2021 and October 2022. Ovarian cysts suspected before the operation or confirmed by postoperative pathology as malignant were excluded, and vaginal infection was a contraindication for vNOTES. TU-LESS or vNOTES was performed according to the patient's wishes. Each patient who chooses vNOTES should be evaluated in detail before the operation, and the vNOTES approach should be avoided in one of the following cases (23, 24): suspected as malignant tumor before the operation, vaginal infection, highly suspected of severe pelvic adhesions, and the lesion location beyond the scope of the instrument of vNOTES. The exclusion criteria of malignant tumors are systematically based on physical signs, imaging, and serum blood tests, which are mainly according to some international expert consensuses (25, 26). Before the surgery, each patient was informed of the surgical risks (i.e., bladder, ureter, and rectum injuries) and benefits, and then signed written informed consent.

### Data collection

Information on all patients was collected from hospital databases, including patient age, body mass index (BMI), the maximum diameter of the cyst, previous pregnancy and abdominal surgery, surgical location, the total time to surgery (i.e., from cutaneous incision to closure), blood loss quantified by subjective visual quantification (27, 28), simultaneously conducting other surgeries, intraoperative complications (i.e., bladder, bowel, and vascular injury), conversion to another surgical procedure, perioperative decrease in serum hemoglobin, postoperative exhaust time, postoperative fever (i.e., any oral temperature≥ occurrence of 38.0°C ≥24 h postoperatively), hospital stay, and postoperative complications. All patients underwent an outpatient review 1 month postoperative to assess postoperative recovery and clinical data.

## Standard operating procedures for vNOTES and TU-LESS

### Preoperation

All surgeries were performed under general anesthesia, and the patient is placed in the bladder lithotomy position. To prevent infection, 1 g of cefmetazole is administered intravenously 30 min before the procedure. The vaginal and perineal areas are repeatedly disinfected with iodophor, and Foley catheters are inserted for all patients.

### Intraoperation

In the TU-LESS group, an incision is made 2 cm at the umbilicus. Then, multiple instrument access ports are inserted through the incision (Beijing Aerospace Cardi Technology Development Institute, HK-TH-60.4TY). In the vNOTES group, an incision was made 1.5 cm at the posterior cervical vault. The operating platform is still built with multiple instrument access ports.

The following steps are the same for both groups. Pneumoperitoneum was generated by CO_2_ blowing up to 14 mmHg and visualized using a 10 mm 30-degree rigid laparoscope (Karl Storz GmbH & Co. KG, Tuttlingen, Germany). After separating the ovarian cyst from the surrounding tissue, part of the cortex along the long axis of the cyst was cut off using scissors to separate the cortex from the cyst wall. Once the cyst was removed, the remaining ovarian tissue was sutured with an absorbable thread, and an oophoroplasty was performed.

In the following situations, for example intraoperative damage to large vessels or important organs, bleeding volume > 500 ml, change the surgical method; vNOTES was converted to transabdominal single-port laparoscopic surgery, while single-port laparoscopic surgery was converted to multiport surgery. In the event of any life-threatening vascular injury, open surgery was performed.

Peritoneal adhesions were assessed and classified according to the Nair scoring system. Adhesions are divided into four degrees according to the degree of adhesion between the two viscera and viscera and abdominal wall. Abdominal and vaginal wounds are closed with 2–0 absorbent sutures and 2–0 barbed absorbable sutures, respectively. In the TU-LESS and vNOTES groups, drainage tubes were not routinely placed.

## Standard day-care procedure

### Preadmission

Selection of suitable patients for the day-care surgery in the outpatient clinic. Exclude those who are suspected of severe pelvic adhesions, malignant tumors, or are not suitable for the day-care surgery.

Conduct preoperative testing in the outpatient setting (including blood examination, tumor markers, pretransfusion antibody screen, coagulation function, urination and leucorrhea routine, human chorionic gonadotropin, electrocardiogram, chest radiograph, and gynecological, abdominal, and urological ultrasounds).

Conduct preoperative evaluation, including the patient's general condition and anesthesia evaluation.

Inform patients of the specific admission time and precautions, such as 8-h fasting and water deprivation before admission, and the complete day-care procedure.

### Hospitalization

The surgery was performed after completing the preoperative evaluation of the patient. After the surgery, the catheter is pulled out immediately, and regular postoperative nursing is carried out.

### Post-discharge

Assess whether the patient meets the discharge criteria (i.e., usually discharged the morning after surgery). If there are any postoperative complications, such as fever, nausea, or vomiting, the patient is discharged on the third day after treatment and observation.

Ensure that patients and their families understand postoperative care and provide written precautions and follow-up arrangements.

Provide postoperative support and perform regular postoperative follow-ups (i.e., on the 7th day and 1 month after surgery).

### Statistical analysis

All statistical analyses were performed using SPSS version 25.0 (IBM, Armonk, NY, USA). Continuous variables are expressed as means and standard deviations and analyzed using the Student's *t*-test, the corrected Student's t-test, one-way ANOVA, or a non-parametric test. Categorical variables are expressed as counts and percentages and analyzed using either the chi-square or Fisher's exact test. Multivariable linear regression analysis was used to assess the influencing factor of intraoperative bleeding, operative time, exhaust time, and postoperative 6-h pain score. Covariates were selected according to the different variables in the univariate analysis and factors reported in previous studies that would affect the dependent variable. All tests were double-tailed; a *p*-value of <0.05 was considered statistically significant.

## Results

**Figure 1:**
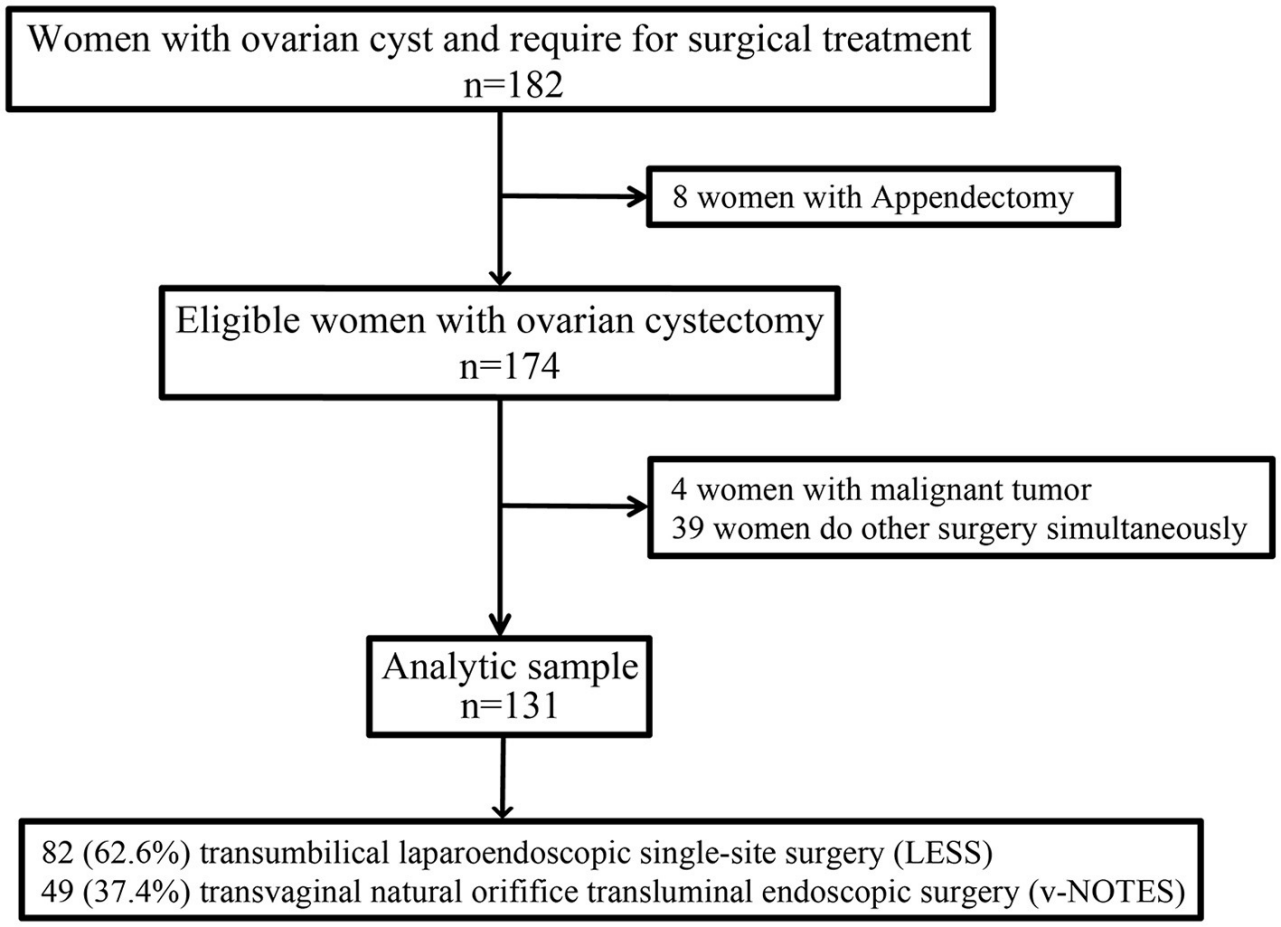
Selection process for this study.

The selection process of the study population is shown in Figure 1. A total of 182 patients with ovarian cysts from our hospital were initially recruited. After excluding patients who had simultaneous surgery and malignant tumor, the final analysis included 131 patients; of which, 82 (62.6%) underwent LESS and 49 (37.4%) underwent vNOTES. Noteworthy, four patients were excluded due to final malign pathology. The characteristics of the patients are presented in Table 1. The average age of patients at recruitment, BMI, and a maximum diameter of cysts was 32.54 ± 7.00 years, 21.59 ± 2.94 kg/m^2^, and 5.19 ± 1.80 cm, respectively. Among these patients, 53 (40.5%) had undergone abdominal surgery, 7 (5.3%) had undergone bilateral ovarian cysts, and 52 (39.7%) had undergone chocolate cysts.

**Table 1:** Description of the patients demographic characteristics and operation types.

**Variables**	**Total**
Patients	131
Age	32.54 ± 7.00
BMI (kg/m^2^)	21.59 ± 2.94
Max diameter of cyst (cm)	5.19 ± 1.80
History of abdominal surgery	53(40.5%)
D & C artificial abortion	0.67 ± 1.08
**Cyst type**	
Simple cyst	48 (36.6%)
Chocolate cyst	52 (39.7%)
Teratoma	31 (23.7%)
Bilateral ovarian cyst	7 (5.3%)
**Myomectomy type**	
Laparoendoscopic single-site surgery (LESS)	82 (62.6%)
V-NOTES	49 (37.4%)

Further analysis of perioperative data showed that there were no significant differences between the two groups for age, BMI, abdominal surgery history, maximum cyst diameter, operation time, intraoperative bleeding, hospital stay, and postoperative complications. In vNOTES, this group had less postoperative exhaust time, a lower postoperative 6-hour pain score, and a lower incidence of analgesic drug use, with a higher surgical conversion incidence. During the postoperative follow-up (1 week and 1 month after surgery), four patients were found to have complications: one patient with febrile, one patient with anemia and transfusion, and one patient with poor wound healing in the LESS group, while one patient with febrile in the vNOTES group. All four complications occurred within 1 week after surgery and were cured in the outpatient department without re-operation (Table 2).

**Table 2:** Description of the patient characteristics by cystectomy types.

**Variables**	**LESS**	**v-NOTES**	***p*** **-value**
**Patients**	***N*** = **82**	***N*** = **49**	
Age (year)	32.74 ± 7.31	32.20 ± 6.51	0.671^a^
BMI (kg/m^2^)	21.36 ± 2.99	21.95 ± 2.85	0.285^a^
History of abdominal surgery	33(40.2%)	20 (40.8%)	0.948^b^
Max diameter of cyst (cm)	6.62 ± 1.59	6.44 ± 1.46	0.065^a^
Pelvic adhesion	9(11.0%)	5(10.2%)	0.838^c^
Bilateral ovarian cyst	3(3.7%)	3(3.7%)	0.424^c^
D&C artificial abortion	0.71 ± 1.13	0.61 ± 1.01	0.629^a^
**Operative information**			
Procedure time (min)	95.47 ± 37.48	86.95 ± 52.03	0.239^a^
Bleeding volume (ml)	40.25 ± 58.24	35.20 ± 42.15	0.425^a^
Surgical conversion	0 (0%)	4 (8.9%)	0.013^c^
**Post-operative information**			
Hemoglobin difference (g/L)	13.71 ± 9.23	15.65 ± 9.43	0.281^a^
Hospital stay (day)	1.05 ± 0.31	1.04 ± 0.20	0.873^a^
Exhaust time (hour)	9.01 ± 7.52	7.14 ± 7.75	0.043^a^
Pain scores (6 h after surgery)	1.41 ± 0.74	1.06 ± 0.91	0.026^a^
Complications	3 (3.7%)	1 (2.0%)	1.000^c^
Pain medications	24 (29.3%)	3 (6.1%)	0.009^c^

a Average and standard deviation. Student's *t*-Test.

b Number (percentage). Chi-squared Test.

c Number (percentage). Fisher Exact Test.

**Figure 2:**
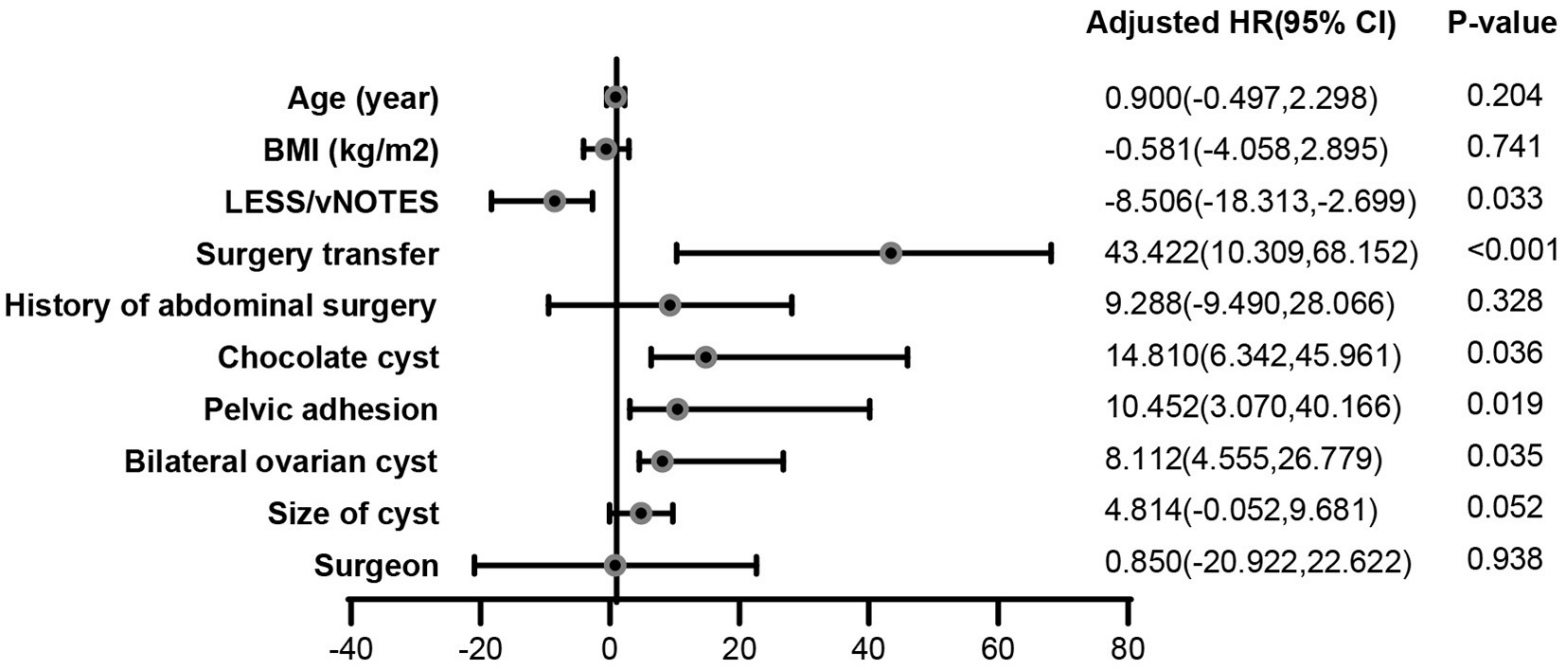
Impact of surgical characteristics on operation duration. The multiple linear regression analysis showed that the operation duration was correlated with the operation approach, surgical conversion, chocolate cyst, bilateral cyst, and pelvic adhesion. The surgical conversion, chocolate cyst, bilateral cyst, and pelvic adhesion increased the operation duration by ~43 (95% confidence interval [CI]: 10.309, 68.152, *p* < 0.001), 15 (95% CI: 6.342, 45.961, *p* = 0.036), 10 (95% CI: 3.07, 40.166, *p* = 0.019), and 8 (95% CI: 4.555, 26.779, *p* = 0.035) min, respectively. Interestingly, vNOTES decreased the operation duration by ~8.5 min (95% CI: −18.313, −2.699, *p* = 0.033).

The operation duration reflects the effectiveness of the surgery. The multiple linear regression analysis showed that the operation duration was correlated with the operation approach, surgical conversion, chocolate cyst, bilateral cyst, and pelvic adhesion. The surgical conversion, chocolate cyst, bilateral cyst, and pelvic adhesion increased the operation duration by ~43 (95% confidence interval [CI]: 10.309, 68.152, *p* < 0.001), 15 (95% CI: 6.342, 45.961, *p* = 0.036), 10 (95% CI: 3.07, 40.166, *p* = 0.019), and 8 (95% CI: 4.555, 26.779, *p* = 0.035) min, respectively. Interestingly, vNOTES decreased the operation duration by ~8.5 min (95% CI: −18.313, −2.699, *p* = 0.033) (Figure 2).

Intraoperative blood loss is an important measure of the safety of surgery. The multiple linear regression analysis showed that the amount of intraoperative bleeding was positively correlated with surgical conversion, chocolate cyst, bilateral cyst, pelvic adhesion, and operation time. The surgical conversion, chocolate cyst, and bilateral cyst increased intraoperative bleeding volume by ~40 (95% CI: 25.023, 85.039, *p* = 0.027), 26 (95% CI: 5.013, 47.678, *p* = 0.016), and 33 (95% CI: 10.276,76.816, *p* = 0.023) ml, respectively. Meanwhile, the bleeding volume increased by ~1.7 ml when the duration of surgery increased by 1 min (95% CI: 0.452, 2.856, *p* < 0.001) and 17 ml when pelvic adhesion increased by 1 grade (95% CI: 11.236, 22.716, *p* = 0.014) (Table 3).

**Table 3:** Association between perioperative characteristics and volume of intraoperative bleeding.

**Variables**	**Beta**	**95% CI**	***P*** **-value**	**VIF**
*R*^2^ = **0.393**				
Age (year)	−0.066	(−1.453, 1.322)	0.925	1.225
BMI (kg/m^2^)	0.072	(−3.318, 3.462)	0.966	1.182
Surgical approach	2.481	(−19.092, 24.054)	0.820	1.602
Surgery transfer	40.031	(25.023, 85.039)	0.027	1.130
History of abdominal surgery	−2.036	(−20.773, 16.702)	0.830	1.200
Chocolate cyst	26.345	(5.013, 47.678)	0.016	1.528
Pelvic adhesion	16.976	(11.236, 22.716)	0.014	1.096
Bilateral ovarian cyst	33.546	(10.276, 76.816)	0.023	1.076
Duration of surgery	1.654	(0.452, 2.856)	< 0.001	1.154
Size of cyst	−1.176	(−6.011, 3.660)	0.630	1.152
Surgeon	−3.598	(−11.390, 4.193)	0.361	1.229
Preoperative hemoglobin	−0.028	(−0.558, 0.503)	0.918	1.172

BMI, body mass index.

In the vNOTES group, the postoperative exhaust time was significantly shorter than that of the LESS group. The multiple linear regression analysis revealed that the postoperative exhaust time was correlated with surgical approach and duration. vNOTES reduced the postoperative exhaust time by ~85 min (95% CI: −60.320, −110.462, *p* = 0.012), while the exhaust time increased by ~7 min when the duration of surgery increased by 1 min (95% CI: 4.768, 8.462, *p* = 0.041) (Table 4).

**Table 4:** Association between postoperative exhaust time and perioperative characteristics.

**Variables**	**Beta**	**95% CI**	***P*** **–value**	**VIF**
*R*^2^ = **0.207**				
Age (year)	−14.501	(−31.433, 2.430)	0.092	1.262
BMI (kg/m^2^)	−17.237	(−58.756, 24.282)	0.411	1.211
Surgical approach	−85.391	(−60.320, −110.462)	0.012	1.683
Surgery transfer	75.849	(−572.222, 723.92)	0.817	1.184
D&C artificial abortion	55.331	(−50.422, 162.084)	0.301	1.195
History of abdominal surgery	185.128	(−44.659, 414.915)	0.113	1.254
Chocolate cyst	242.828	(−16.322, 501.988)	0.066	1.581
Bilateral ovarian cyst	76.089	(−394.306, 546.484)	0.749	1.210
Pelvic adhesion	−268.048	(−1,345.821, 809.724)	0.622	1.114
Duration of surgery	6.615	(4.768, 8.462)	0.041	1.705
Intraoperative bleeding	−0.209	(−2.931, 2.513)	0.879	1.770
Size of cyst	23.759	(−34.445, 81.962)	0.419	1.162
surgeon	−23.324	(−119.937, 73.288)	0.632	1.289
Pain score (6 h at surgery)	98.552	(−10.880, 207.984)	0.077	1.195
Pain medications	124.715	(−118.924, 368.953)	0.312	1.141
Postoperative complications	239.915	(−362.779, 842.610)	0.431	1.352

BMI, body mass index.

Further multivariate linear regression revealed that the 6-h postoperative pain score was correlated with the operation approach and abdominal surgery history. vNOTES reduced the 6-h postoperative pain score by ~0.9 (95% CI: −1.697, −0.008, *p* = 0.047) and previous abdominal surgery history reduced the score by ~0.6 (95% CI: −1.037, −0.175, *p* = 0.012) (Table 5).

**Table 5:** Association between 6-h pain score postoperatively and perioperative characteristics.

**Variables**	**Beta**	**95% CI**	***P*** **-value**	**VIF**
*R*^2^ = **0.187**				
Age (year)	0.014	(−0.019, 0.047)	0.392	1.292
BMI (kg/m^2^)	0.031	(−0.050, 0.111)	0.451	1.212
Surgical approach	−0.902	(– 1.697, −0.008)	0.047	1.588
Surgery transfer	−0.254	(−1.498, 0.989)	0.685	1.170
D & C artificial abortion	−0.103	(−0.307, 0.101)	0.318	1.195
History of abdominal surgery	−0.606	(−1.307, −0.175)	0.012	1.184
Chocolate cyst	−0.190	(−0.697, 0.316)	0.457	1.618
Bilateral ovarian cyst	−0.101	(−1.009, 0.807)	0.825	1.210
Pelvic adhesion	−0.555	(−2.602, 1.491)	0.591	1.078
Duration of surgery	0.002	(−0.004, 0.007)	0.582	1.722
Intraoperative bleeding	0.001	(−0.005, 0.006)	0.829	1.764
Surgeon	−0.113	(−0.298, 0.072)	0.230	1.271
Postoperative exhaust time	−0.004	(−0.029, 0.009)	0.059	1.196
Postoperative complications	−0.738	(−1.869, 0.392)	0.197	1.275

BMI, body mass index.

## Discussion

Vaginal natural orifice transluminal endoscopic surgery has advantages, such as reduced postoperative pain, shorter exhaust time and hospital stay, and absence of postoperative scar, which aligns with the concept of the day-care surgery (12, 29). In this retrospective preliminary study, we compared the perioperative data of vNOTES for ovarian cystectomy in the day-care procedure with those of single-port laparoscopic surgery. We also outlined the advantages and disadvantages of LESS and vNOTES for ovarian cystectomy in the day-care procedure, which could lay a theoretical basis for future vNOTES surgery in a wider area.

In our cohort, there was no significant difference between the vNOTES and LESS groups in terms of intraoperative bleeding volume, operation time, and incidence of postoperative complications, which suggested that ovarian cystectomy *via* vNOTES in the day-care procedure has the same safety threshold as LESS. However, the conversion rate of surgery, which indicates the effectiveness of surgery, was significantly different between the two groups. In the vNOTES group, the surgical conversion rate was higher than that previously reported, which was attributed to the presence of pelvic adhesions. It was difficult to penetrate the pelvic cavity during the surgery. If vNOTES was continued, there is a risk of damage to adjacent organs; thus, it was switched to LESS. During LESS, the posterior wall of the uterus was tightly adhered to the pelvis, sealing the pelvis. Notably, the four patients had chocolate cysts. Due to the timely transit during surgery, no damage to the adjacent organs occurred, and there was no significant difference in the complication rate between the two groups. Therefore, surgeons should conduct a more comprehensive preoperative evaluation on patients who choose to undergo vNOTES, such as those with multiple abdominal surgeries and chocolate cysts (23, 30). Our study also included careful inquiry of dysmenorrhea history and gynecological physical examination to assess uterine activity and tenderness of the surface nodules of the sacral ligaments (24, 31). Moreover, multi-parameter scores for ovarian tumors (such as ESGO/ISUOG/IOTA/ESGE) (32) may be used to evaluate endometriosis to find severe adhesion before surgery. In addition, vaginal ultrasound can also be used to evaluate the sliding of the uterus on the anterior wall of the rectum in real-time. If severe pelvic adhesion is suspected after evaluations, vNOTES should be avoided (13, 33–35).

The difficulty of vNOTES lies in the establishment process (i.e., the approach and lesion exposure processes). The previous study showed that the operation duration is longer in vNOTES than that in LESS (36–38). However, in this cohort, the vNOTES group had a shorter operating duration, which may be due to the advantage of the visual field. Ovarian cysts, especially teratomas, can be directly assessed *via* the incision of vNOTES; therefore, we consider that vNOTES has an advantage over LESS for ovarian cystectomy. Consistent with the study of Giovanni Buzzaccarini et al., the new target of clinical trials should be to assess the appropriateness of vNOTES in selected populations, with the aim of maximizing the potential benefits of this technique compared to other approaches (39). For obese women and/or for women with large uteri, vNOTES may be particularly effective and safe (40). Moreover, for giant teratoma, specimen retrieval still represents an issue. Vaginal muscles have better ductility, making it easier to take out some large specimens, which may be another advantage of vNOTES (41, 42).

Consistent with previous studies, we also found that the vNOTES group had shorter postoperative exhaust times than that of the LESS group (43, 44), which may be due to the following reasons. First, vNOTES surgery is performed in the pelvis and has little effect on the upper abdomen. Second, before surgery, the small intestine is pushed to the true pelvic level. Therefore, the surgical instruments do not repeatedly contact the intestine, reducing irritation to the intestine. In addition, blood collects between the endoscopic body and the target area due to the upward viewing angle of surgery. Furthermore, the blood is constantly washed during the procedure to ensure clear vision. The postoperative residual blood volume in the abdominal cavity is significantly reduced, chemical irritation and inflammatory factors are reduced, and bowel function recovers faster (45–47).

Moreover, the vNOTES group experienced milder postoperative pain than that of the LESS group (33, 48, 49), which may be due to the vaginal fornix being innervated by visceral nerves and is not sensitive to pain. vNOTES also avoids damaging the abdominal wall, which occurs in LESS due to trocar puncture. Furthermore, the operation time of vNOTES was relatively shorter than that of LESS for ovarian cystectomy, which reduced the stimulation of the diaphragm by abdominal gas. Milder postoperative pain allows patients to get out of bed earlier, which promotes the recovery of gastrointestinal function after surgery, resulting in earlier postoperative exhaust.

Based on the ERAS concept, the day-care procedure requires surgeons to choose a surgical approach that is less damaging to the patient to ensure less postoperative pain, shorter exhaust time, faster recovery, and safer discharge within 24 h. Moreover, scarless surgery is the current trend of gynecological surgery; the vNOTES approach conceals the incision, avoids abdominal damage, and reduces the occurrence of incision hernia, which invokes psychologically minimal invasive effects and allows for the rapid recovery of patients.

The strength of this study is that it specifically studies the population and standard operating procedures for vNOTES. Participants were screened using rigorous inclusion and exclusion criteria. Patients who had other surgeries simultaneously or malignant ovarian cysts were excluded. This study compared the comprehensive perioperative data of the two latest surgical methods, LESS and vNOTES, for ovarian cystectomy, and preliminarily confirmed the effectiveness and safety of vNOTES. In addition, all patients underwent an outpatient review 1-month postoperatively to assess postoperative recovery and obtain complete clinical data, resulting in a relatively comprehensive study design. Moreover, our hospital has implemented vNOTES since 2018, and there are nearly 2,000 vNOTES cases per year in the last 2 years; thus, all operations were performed according to a standardized surgical procedure.

The pilot study enhances our understanding of LESS and vNOTES for ovarian cystectomy. However, our study had several limitations. First, the sample size of this study was relatively small compared to similar studies of multiport and LESS. Second, this study is retrospective, and vNOTES has been widely used in gynecology for only 5 years. Prospective follow-up of patients after ovarian cystectomy can provide further insight into short- and long-term complications and the potential impact of vNOTES on sexual function, pregnancy, and vaginal delivery. Therefore, a large-scale multicenter study involving more patients and different types of gynecological diseases is needed to further promote the widespread use of vNOTES.

## Conclusion

Vaginal natural orifice transluminal endoscopic surgery was equally safe and effective for ovarian cystectomy compared to that of LESS. vNOTES aligned with the concept of the day-care procedure due to its reduced postoperative pain, shorter exhaust time, and absence of scarring. However, surgeons should conduct a comprehensive preoperative evaluation and exclude patients who are suspected to have severe pelvic adhesions.

## Data availability statement

The raw data supporting the conclusions of this article will be made available by the authors, without undue reservation.

## Author contributions

YLin, YJ, and XG conducted the study and provided funding resources. XL and AX analyzed the data and drafted the manuscript. HW, YLiu, LW, JL, JY, ZY, JZ, LH, TL, and YLi critically revised the manuscript. All authors have accepted responsibility for the entire content of this submitted manuscript and approved submission.
